# The effects of high dose interferon-β1a on plasma microparticles: Correlation with MRI parameters

**DOI:** 10.1186/1742-2094-8-43

**Published:** 2011-05-09

**Authors:** Mary Lowery-Nordberg, Erin Eaton, Eduardo Gonzalez-Toledo, Meghan K Harris, Kathrine Chalamidas, Jeanie McGee-Brown, Chaitanya V Ganta, Alireza Minagar, David Cousineau, J Steven Alexander

**Affiliations:** 1 Department of Pathology LSU Health Sciences Center-Shreveport, 1501 Kings Highway, Shreveport, LA 71130-3932, USA; 2 Department of Molecular and Cellular Physiology, 1501 Kings Highway, Shreveport, LA 71130-3932, USA; 3 Department of Radiology, 1501 Kings Highway, Shreveport, LA 71130-3932, USA; 4 Department of Neurology, 1501 Kings Highway, Shreveport, LA 71130-3932, USA; 5 Feist-Weiller Cancer Center, LSU Health Sciences Center-Shreveport, 1501 Kings Highway, Shreveport, LA 71130-3932, USA

## Abstract

**Objectives:**

We previously reported a correlation between levels of microparticles carrying CD31 (PMP ^CD31+^) and disease activity in MS. However, the effects of long term (12 month) treatment with high dose, high frequency interferon-β1a (Rebif™) on plasma levels of PMP^CD31+^, PMP^CD146+^, and PMP^CD54+ ^and MRI measures of disease activity have not yet been assessed.

**Methods:**

During this prospective 1-year study, we used flow cytometry to measure changes in plasma microparticles (PMP) bearing CD31 (PMP^CD31+^), CD146 (PMP^CD146+^), and CD54/ICAM-1 (PMP^CD54+^) in 16 consecutive patients with relapsing-remitting MS (RRMS) before and after 3, 6, and 12 months of subcutaneous therapy with interferon-beta1a (44 micrograms, 3X weekly). At each visit, clinical exams and expanded disability status scale (EDSS) scores were recorded.

**Results:**

Plasma levels of PMP^CD31+^, and PMP^CD54+ ^were significantly reduced by treatment with IFN-β1a. PMP^CD146+ ^appeared to decrease only at 3 months and did not persist at 6 and 12 months (p = 0.0511). In addition, the decrease in plasma levels of PMP^CD31+ ^and PMP^CD54+ ^levels at 12 months were associated with a significant decrease in the number and volume of contrast enhancing T1-weigthed lesions.

**Conclusion:**

Our data suggest that serial measurement of plasma microparticles (PMP), particularly in the initial stages of MS (when neuro-inflammatory cascades are more intense), may serve as reliable and reproducible surrogate markers of response to IFN-β1a therapy for MS. In addition, the progressive decline in plasma levels of PMP^CD31+ ^and PMP^CD54+ ^further supports the concept that IFN-β1a exerts stabilizing effect on the cerebral endothelial cells during pathogenesis of MS.

## Introduction

Multiple sclerosis (MS) is an immune-mediated neurodegenerative disease of the human central nervous system (CNS) characterized clinically by a relapsing-remitting course. Neuropathologically MS manifests with development of demyelinating lesions which affect both gray and white matter [1,2] disruption of the blood brain barrier (BBB) and transendothelial migration of lymphocytes and macrophages across inflamed CNS endothelial monolayers appear to be among the earliest CNS and spinal cord abnormalities in MS [3]. Activation of the cerebral endothelial cells and their binding with activated leukocytes are early crucial steps in creation of MS demyelinating lesions produced by immune cells [4,5].

Cerebral endothelial cells, the main components of the BBB, contain tight and adherens junctions which create a highly impenetrable and impermeable anatomic-physiologic barrier against circulating plasma neurotransmitters, cytokines, formed blood elements and soluble and insoluble molecular components of the circulating blood. Upon activation by pro-inflammatory cytokines such as IFN-γ or TNF-α, released by activated T-lymphocytes, endothelial cells shed small fragments of their membranes, known as endothelial microparticles (PMP) [6]. These fragments contain some of the surface adhesion molecules and other endothelial markers of their parent cells [7], including, but not limited to, platelet-endothelial cell adhesion molecule (PECAM-1/CD31), human endothelial marker CD146, and intercellular adhesion molecule (ICAM-1/CD54). Therefore while it is difficult to evaluate inflammatory endothelial markers in MS in situ, microparticles may provide a remote 'snapshot' of the surface of inflamed endothelium and provide information on the extent of platelet and leukocyte activity in MS.

We previously reported elevated plasma levels of PMP in MS patients and demonstrated that the plasma levels of PMP^CD31+ ^may reflect acute endothelial injury with positive association with presence of contrast enhancing lesions [3]. Additionally, we reported that various sub-species of PMP form complex with different leukocytes and by formation of such complexes, they promote the inflammatory process by facilitating transendothelial migration of the leukocytes [6]. In the current study, we hypothesized that prospective serial measurement of three sub-species of PMP CD31^+^, PMP^CD146+^, and PMP^CD54+ ^may reflect disease activity and serve as a surrogate marker of therapeutic response to high dose, high frequency interferon-β1a. We also assessed the correlation among these sub-species of PMP and MRI parameters.

## Methods

During this one year prospective study, a cohort of 16 patients who met the Poser et al. criteria [8] for clinically definite MS, were studied. The clinical study was approved by Louisiana State University Health Sciences Center-institutional review board and all patients provided informed consent. For at least three months prior to initiation of treatment with IFN-β1a, none of the subjects had received any immunosuppressive treatment or corticosteroids. All study subjects underwent neurological examination at the study entry prior to initiation of treatment with IFN-β1a and at 3, 6, and 12 months after initiation of treatment. At each visit, their expanded disability status scale (EDSS) scores were determined. Peripheral venous blood specimens (baseline) were collected prior to initiation and within the first 15 hours of treatment with IFN-β1a and at months 3, 6, and 12 after treatment initiation. The study subjects were treated with IFN-β1a 44 micrograms ug subcutaneously three times weekly. Because therapy cannot be withheld from MS patients, this study did not contain an arm consisting of untreated MS patients.

## Measurement of PMP using flow cytometry

Using a 21 gauge needle, venous blood was obtained in citrate loaded vacutainer tubes. Measurements of plasma PMP were performed within four hours from specimen collection. Briefly, blood specimen was centrifuged at 160 × g for 10 minutes to prepare platelet rich plasma (PRP). Next, the PRP specimen was centrifuged for 6 minutes at 1500 × g to generate platelet poor plasma (PPP). Then, each 25 μl aliquot of PPP was incubated with 2 μl of anti-CD31-PE, 2 μl of anti-CD146, and 2 μl of anti-CD54 at ambient temperature for 20 minutes with gentle shaking (80 rpm). The PMPs in the sample were measured using a Beckman Coulter FC500 MCL flow cytometer system equipped with CXP software (Beckman Coulter, Miami, FL). The sample flow rate and particle detection settings are identical to that described previously [3]. Data collection is based on both multi-parameter and uni-parameter analyses of PMP^CD31+^, PMPCD^146+^, and PMP^CD54+^. To evaluate the possibility that leukocyte adhesion of microparticles might be measured, the anti-leukocyte common antigen CD45 was used in the cocktails. With the exception of whole blood analysis (used as a positive control) negligible CD45^+ ^events were detected in the PPP preparations.

## Statistical analysis

Data were analyzed using Instat (Graphpad software, La Jolla CA) using repeated measures analysis of variance with Dunnett's post-testing to determine time points which differed statistically from starting baseline values. Data are shown as the average ± standard error. Statistical differences in contrast enhancing T1-weighted lesion volumes at t = 0 were compared with t = 12 months using two tailed unpaired students t-test. Microparticle data are shown from t = 0 to 12 months. PMP counts and volumes of contrast enhancing T1-weighted data are also plotted bi-directionally to show separate non-overlapped groupings for PMP^CD54+ ^(ICAM-1) and PMP^CD31+ ^with contrast enhancing T1weighted volumes.

## Magnetic resonance imaging protocol

All study subjects underwent brain MRI by a 1.5 T machine with a standard head coil prior to initiation of therapy with IFN-β1a (Rebif). The neuroimaging protocol consisted of sagittal T1-weighted axial T1- and T2- and proton density weighted images. All MR studies were done after infusion of gadolinium diethylenetriamine pentaacetic acid (Gd). We used post-contrast T1-weighted and T2-weighted axial images for assessment of MS plaques. A neuroradiologist blinded to the subjects' clinical information, interpreted images and measured the volume and number of MS lesions. The second MRI of brain was obtained at month 12 after initiation of treatment with IFN-β1a.

## Results

Subjects with MS had a mean age of 31 years at study entry and had a mean duration of the illness of 14 months. There were 14 females and 2 male in this study. During the course of this study five subjects developed transient elevation of liver enzymes which eventually resolved. Apart from this, no other adverse events occurred as a result of IFN-β1a treatment. During this study we assessed the PMP profiles in MS patients pre- and post treatment with IFN-β1a (Rebif) and found that several but not all classes of plasma endothelial microparticles were altered in MS patients following treatment over 12 months. All data are reported as means ± standard error.

### CD31

**Figure 1:**
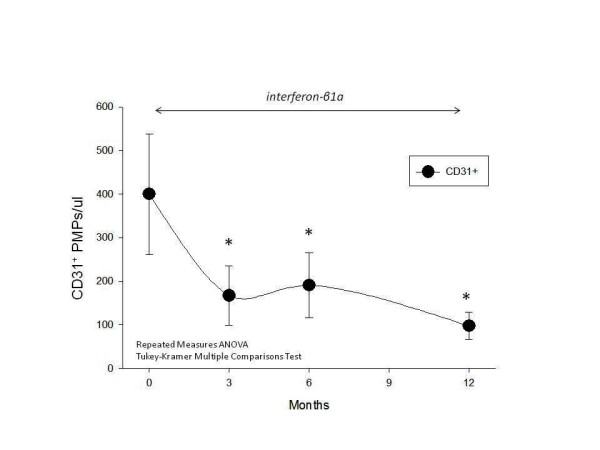
**CD31**. Levels of CD31^+ ^(PECAM-1) microparticles were reduced at 3 and 6 months after IFN-β1a therapy, but did not reach statistical significance until 12 months (* = p < 0.05).

Plasma levels of CD31^+ ^(PECAM-1^+^) microparticles/ul were reduced from a baseline level of 400 ± 138/ul at t = 0 to 167 ± 68/ul at 3 months, and 190 ± 74/ul at 6 months (not statistically significant). Data only reached statistical significance at 12 months when PMP^CD31+ ^were 98 ± 32/ul (Figure 1, * = p < 0.05). This may reflect individual variations in patient's responsiveness to treatment with IFN-β1a which did not become uniform enough to reach statistical significance until the 12^th ^month of treatment. However, comparison of plasma levels of PMP^CD31+ ^at baseline versus one year of treatment with IFN-β1a indicates that this marker shows a significant difference in the quantity of shed PMP into plasma.

### CD146

**Figure 2:**
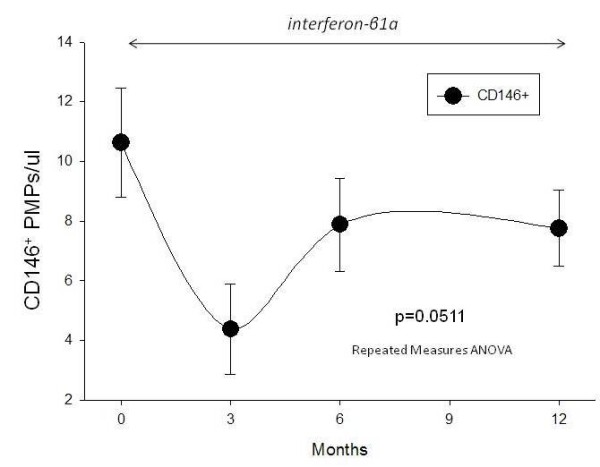
**CD146**. Levels of CD146^+ ^microparticles were marginally statistically significantly reduced after IFN-β1a therapy (p = 0.051). After 3 months of IFN-β1a therapy, CD146+ MPs appeared reduced; however these levels rebounded by 6 and at 12 months.

CD146 also known as melanoma cell adhesion molecule (Mel-CAM) or cell surface glycoprotein 'MUC18' [9] was marginally statistically altered by IFN-β1a treatment (p = 0.0511) (Figure 2). After 3 months of IFN-β1a therapy PMP^CD146+^/ul were reduced from a baseline level (t = 0) of 10.6 ± 1.8/ul, to 4.4 ± 1.5/ul at 3 months; these levels returned to 7.9 ± 1.6/ul by 6 months, and 7.75 ± 1.3/ul at 12 months.

### CD54

**Figure 3:**
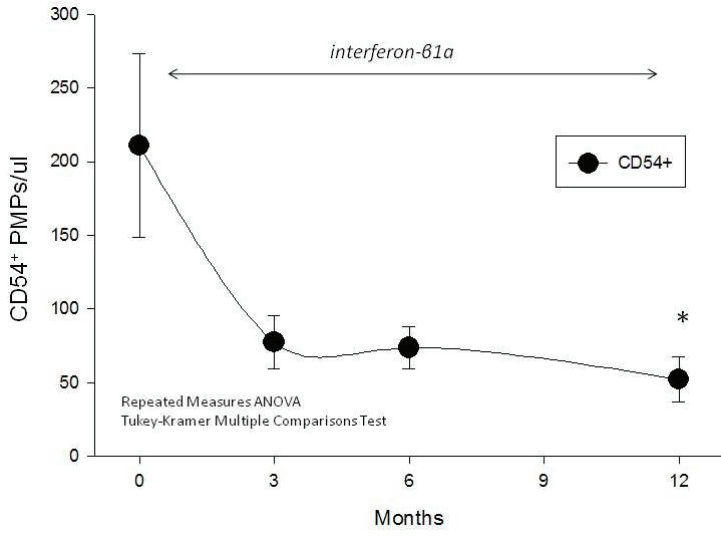
**CD54**. By comparison, CD54+ (ICAM-1+) plasma microparticles were statistically significantly reduced (*, p < 0.05) at 3, 6 and 12 months after IFN-β1a therapy compared to t = 0.

By comparison CD54^+ ^(ICAM-1^+^) plasma endothelial microparticles/ul were reduced from a baseline of 211 ± 62.6/ul at t = 0, to 77.4 ± 18.2/ul at 3 months (* p < 0.05), to 74.7 ± 14.4/ul at 6 months (* p < 0.05) and 52.1 ± 15.2 at 12 months (* p < 0.05) after IFN-β1a therapy compared to t = 0 (Figure 3), suggesting that CD54 represents the most sensitive microparticle marker of disease activity.

**Figure 4:**
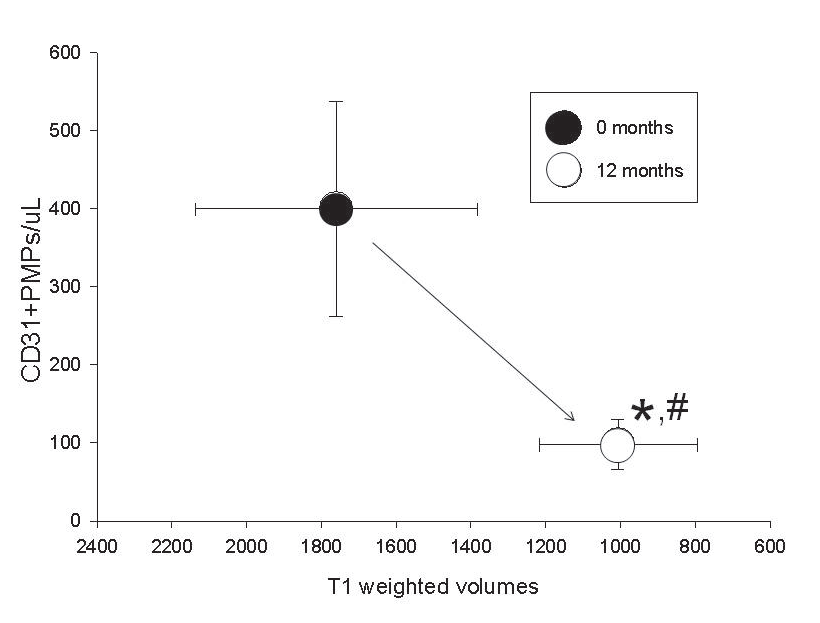
**Correlation between CD31+ EMP and T1 weighted MRI volumes shows that at 12 months both markers were significantly reduced by IFN-β1a therapy compared to t = 0**.

**Figure 5:**
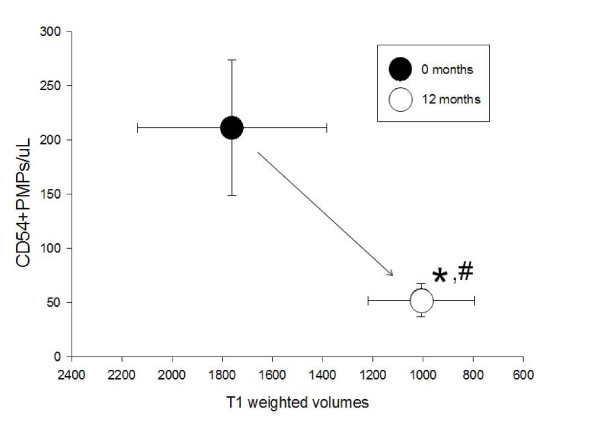
**Correlation between CD54^+ ^EMP and T1 weighted MRI volumes shows that at 12 months both markers were significantly reduced by IFN-β1a therapy compared to t = 0**.

We compared the plasma levels of CD31^+ ^PMP with the volume of contrast enhancing T1-weighted lesions pre- and post treatment with IFN-β1a in order to detect any correlations between these two parameters. Figure 4 shows a bimodal graph correlating plasma levels of PMP^CD31+ ^with the volumes of these lesions. We found that at 12 months both markers were significantly reduced by IFN-β1a therapy. Similarly Figure 5 also depicts the bimodal graph relationship between PMP^CD54+ ^and volumes of contrast-enhancing T1-weighted lesions. Interestingly the magnitude of the reduction in these PMP profiles appears to be similar for both PMP^CD54+ ^and PMP^CD31+ ^when correlated with the volumes of contrast-enhancing T1-weighted lesions. We did not find any significant correlation between the plasma levels of various classes of PMP and the volumes of T2-weighted lesions pre- and post treatment with IFN-β1a.

## Discussion

High dose high frequency IFN-β1a is an FDA-approved treatment for MS which reduces annual relapse rate and improves brain MRI findings. The therapeutic benefits from IFN-β1a have been attributed to may involve effects on immune cells elevation of IL-10 and reduced synthesis of Th1 cytokines endothelial cell adhesion molecules MMPs resulting in lower leukocyte penetration of the CNS parenchyma, antigen presentation by microglia. We observed a decline in the plasma levels of CD31^+ ^microparticles following treatment of MS patients with IFN-β1a. The plasma levels of PMP^CD31+ ^were found to be statistically reduced by IFN-β1a therapy at 3, 6 and 12 months. Because these studies were performed with single staining protocols we cannot specifically designate these particles as 'endothelial', and these must be considered to be a mixture of platelet and endothelial derived microparticles. CD31/PECAM-1 is known to mediate the transendothelial migration of leukocytes [10] in cytokine independent migration. Therefore, shedding of CD31 in microparticles, may represent an adaptive response aimed at limiting availability of CD31 on the endothelial, platelet or leukocyte membranes or alternatively may be another factor which facilitates transendothelial migration of the activated leukocytes. Therefore, a decrease in shedding of PMP^CD31+ ^into plasma following treatment with IFN-β1b may indicate a lessened interaction between activated leukocytes and inflamed underlying endothelium, which in turn translates into stabilization of the blood brain barrier and decrease in the number of contrast-enhancing T1-weighted lesions on brain MRI [3]; [11]. In addition, so-called 'insoluble' pools of CD31 found on microparticles may function as antagonists of leukocyte-bound CD31/PECAM-1 which functionally limits CD31 access, leading to reduced binding and transmigration.

Turning to another endothelial marker, PMP^CD146+^, we observed the effect of treatment of IFN-β1a on the plasma levels of this biological marker. CD146 is abundantly expressed on the surface of brain endothelial cells where it may act as a specific functional ligand for Th17 cells [12]. CD146 is also expressed on the surface of a subpopulation of activated T-cells [13] and mediates lymphocyte-endothelial adhesion. CD146+ microparticles were nearly statistically significantly reduced over 12 months of therapy with IFN-β1a. This change in CD146^+ ^microparticle expression appeared at 3 months of therapy, and was not observed at 6 or 12 months. CD146, is also known as MUC18, which is considered to be a marker of endothelial cells, however it can also be found on a subset of T- and B-lymphocyte, NK cells [14], pericytes [15] and circulating endothelial cells [16]. Plasma levels of endothelial derived 'soluble' CD146 were inversely correlated with congestive heart failure [17], it is not clear whether how transient reductions in CD146 PMPs might be interpreted. Plasma levels of PMP^CD146+ ^cannot yet predict a long term stabilizing effect of IFN-β1a on cerebral endothelial cells.

During the course of this prospective study, we also assessed the plasma levels of PMP^CD54+ ^prior to treatment with IFN-β1a and at timed intervals (3, 6 and 12 months) after treatment. PMP^CD54+ ^appears to mainly represent endothelial microparticles-derived ICAM-1 (CD54), but ICAM-1 can also be expressed by macrophages and T-cells. CD54 is constitutively expressed on the surface of endothelial cells [19], and its' expression is significantly upregulated by Th1 cytokines (TNF-α, IL-1b, IFN-γ) which are known to be elevated during MS exacerbations [19]; [20]. While some studies have shown elevation of soluble forms of ICAM-1 (CD54) during active MS disease, [21] our study shows that levels of insoluble microparticles bearing CD54 in MS disease are significantly reduced by IFN-β1a therapy at 3, 6 and 12 months. In addition, previously Jy et al. [6] demonstrated that PMP^CD54+ ^formed conjugates with monocytes and facilitated their migration through the endothelial cells monolayers and may represent an important mechanism supporting the penetration of immune cells into the CNS parenchyma in MS.

The correlation of plasma levels of PMP^CD31+ ^and volumes of contrast-enhancing T1-weighted lesions on brain MRI is presented as a bimodal graph in Figure 4. We found that at 12 months both markers (PMP^CD31+ ^and T1-weighted volumes) were significantly reduced by IFN-β1a therapy. Similarly, Figure 5 also depicts the bimodal graph relationship between PMP^CD54+ ^and volumes of contrast-enhancing T1-weighted lesion on brain MR images. Interestingly, when plotted in this manner, the magnitude of the reduction in these profiles appears to be similar for both PMP^CD31+ ^and PMP^CD54+ ^with volumes of the contrast-enhancing T1-weighted lesions. Whether and how these parameters are mechanistically linked to therapeutic benefit of IFN-b1α in MS is not clear but lower CD54 appears to reflect decreased endothelial activation. Because endothelial CD31/PECAM-1 is not as dramatically altered by exposure Th1 cytokines, as CD54 is for example [22], diminished CD31 levels suggest stabilization of membrane integrity and reduced platelet activation both of which could contribute to therapeutic benefit in MS.

## Conclusions

Collectively, the results of our study lend support to the concept that administration of IFN-β1a to MS patients is associated with stabilization of the cerebral endothelial cells. This is reflected by concurrent decreases in the plasma levels of both PMP^CD31+ ^and PMP^CD54+ ^as well as volumes of contrast-enhancing T1-weighted lesions. We recommend that, at least during the early stages MS pathogenesis, measurement of certain sub-populations of plasma endothelial microparticles may serve as a reliable biological marker for disease activity and response to treatment with beta-interferons.

## Competing interests

The authors declare that they have no competing interests.

## Authors' contributions

MLN - Helped to conceive study and directed MP flow analysis work, EE coordinated patient sample collection and interacted with AM, EGT carried out all patient MRI analyses, MKH interviewed, consented and collected patient specimens, KC collected clinical patient information and coordinated between AM, MKH and EGT, JMB collected patient clinical information data and coordinated between AM, MKH and EGT, CVG interpreted data, statistics and helped write manuscript, AM helped conceive the study and helped to write manuscript, DC worked on MP flow cytometry and helped perform statistics, JSA helped to conceive study, interpret data, statistics, figures and helped to write manuscript. All authors read and approved the final manuscript.
